# Tissue- and Plasma-Specific MicroRNA Signatures for Atherosclerotic Abdominal Aortic Aneurysm

**DOI:** 10.1161/JAHA.112.000745

**Published:** 2012-10-25

**Authors:** Keiwa Kin, Shigeru Miyagawa, Satsuki Fukushima, Yukitoshi Shirakawa, Kei Torikai, Kazuo Shimamura, Takashi Daimon, Yukio Kawahara, Toru Kuratani, Yoshiki Sawa

**Affiliations:** 1 Department of Cardiovascular Surgery, Osaka University Graduate School of Medicine, Suita, Osaka, Japan K.K., S.M., S.F., Y. Shirakawa, K.T., K.S., T.K., Y. Sawa); 2 Laboratory of RNA Function, Osaka University Graduate School of Medicine, Suita, Osaka, Japan (Y.K.); 3 Department of Biostatistics, Hyogo College of Medicine, Nishinomiya, Hyogo, Japan (T.D.)

**Keywords:** abdominal aortic aneurysm, aneurysm, atherosclerosis, miRNA

## Abstract

**Background:**

Atherosclerotic abdominal aortic aneurysm (AAA) is a progressive, gradual aortic rupture that results in death in the absence of surgical intervention. Key factors that regulate initiation and progression of AAA are unknown, making targeted interventions difficult. MicroRNAs play a fundamental role in atherosclerosis, and atherosclerotic coronary artery disease is characterized by tissue- and plasma-specific microRNA signatures. However, little is known about microRNAs involved in AAA pathology. This study examined tissue and plasma microRNAs specifically associated with AAA.

**Methods and Results:**

AAA and normal wall tissues were sampled from patients undergoing AAA repair (n=13; mean age, 68±6 years) and aortic valve replacement surgery (n=7; mean age, 66±4 years), respectively. MicroRNA expression was assessed by high-throughput microRNA arrays and validated by real-time polymerase chain reaction for individual microRNAs that showed significant expression differences in the initial screening. MicroRNAs related to fibrosis (miR-29b), inflammation (miR-124a, miR-146a, miR-155, and miR-223), and endothelium (miR-126, let-7 family members, and miR-21) were significantly upregulated in AAA tissue. Significant negative correlations were seen in expression levels of monocyte chemoattractant protein-1 and miR-124a, -146a, and -223; tumor necrosis factor-α and miR-126 and -223; and transforming growth factor-β and miR-146a. Expression of microRNAs, such as miR-29b, miR-124a, miR-155, and miR-223, that were upregulated in AAA tissue was significantly reduced in plasma of patients with AAA (n=23; mean age, 72±9 years) compared to healthy controls (n=12; mean age, 51±11 years) and patients with coronary artery disease (n=17; mean age, 71±9 years).

**Conclusions:**

The expression of some microRNAs was specifically upregulated in AAA tissue, warranting further studies on the microRNA function in AAA pathogenesis and on the possibility of using a microRNA biomarker for AAA diagnosis.

## Introduction

Atherosclerosis is a global concern and generates an array of lethal pathologies in the elderly. Abdominal aortic aneurysm (AAA) is a common atherosclerosis-related disease that eventually leads to aortic rupture and death if not treated by graft replacement surgery or covered stent implantation, which are the established armamentarium against this pathology. AAA is a progressive disease characterized by aortic wall atherosclerosis, in which vascular smooth muscle cells (VSMCs) and infiltrating monocytes/macrophages are activated to release a variety of factors, including matrix metalloproteinases, angiotensin II, tumor necrosis factor (TNF)-α, interleukin-1β, interleukin-6, and interferon-γ, consequently generating a chronic inflammatory state. However, key factors that regulate initiation or progression of these processes, and thus potential therapeutic targets, are poorly studied.

MicroRNAs (miRNAs), which are small noncoding RNAs that posttranscriptionally regulate gene expression,^1^ play a major role in several biological and pathological processes, including cellular activities,^2–5^ atherosclerosis,^6^ and cancer.^7^ Initiation and progression of some atherosclerosis-related cardiovascular diseases are regulated at least partially by local miRNA signatures, which therefore could be therapeutic targets; however, expression patterns and biological roles of miRNA in AAA pathogenesis have not been elucidated. In addition, the circulating plasma miRNA signature might be correlated with the local miRNA expression signature of a specific pathology and thus can be used clinically as a biomarker.^8^ Although it recently was suggested that some specific miRNA signatures might be present in the plasma of patients with coronary artery atherosclerotic disease, little is known about AAA-specific plasma miRNA signatures.

In the present study, we hypothesized that atherosclerosis-related miRNAs are upregulated or downregulated in AAA tissue and in plasma of patients with AAA, as compared to the normal population. Therefore, we comprehensively analyzed miRNA expression in human AAA tissue by using oligonucleotide microarray and real-time quantitative polymerase chain reaction (PCR) to identify candidate miRNAs, and we further confirmed their roles in AAA pathogenesis. Moreover, we examined relationships between blood circulatory miRNA expression and pathological changes of the aorta.

## Methods

This study was approved by the institutional ethics committee (reference No. 09277). All members of the study cohort gave written informed consent before entering the study. All evaluations were carried out in a blinded manner. The authors had full access to the data and take full responsibility for the integrity of the data. All authors have read and agreed to the manuscript as written.

### Cohort Background and Characteristics and Sample Collection

We collected full-thickness aortic wall samples from the anterior wall of the infrarenal AAA (aneurysmal area) of 13 patients undergoing elective open AAA repair. We also sampled ascending aorta without visible atherosclerotic changes (normal aorta) as age- and sex-matched controls from 7 patients undergoing aortic valve replacement surgery (Table 1). Preoperative characteristics, including hypertension, diabetes mellitus, and “on-statin” medication status, were not different between the 2 groups. Aortic wall samples were processed promptly for RNA and histological analysis. In addition, we collected blood samples from 23 patients with AAA; 12 healthy volunteers without atherosclerotic disease or inflammatory disorders, as a control group; and 17 patients with coronary artery disease (CAD), as a disease control group (Table 2). Age, hypertension, and “on-statin” status were not similar among the 3 groups. Patients with impaired ejection fraction, heart failure, unstable CAD, or acute myocardial injury were excluded. General exclusion criteria were a known history of leucopenia, thrombocytopenia, or severe hepatic or renal dysfunction, as well as evidence for inflammatory or malignant disease. The plasma was separated promptly and stored at −80°C until use.

**Table 1.** Patient Characteristics (Tissue Samples)

	AAA (n=13)	Normal (n=7)
Age, y	68.3±6.1	66.4±4.1
Male, n (%)	10 (77)	5 (71)
Background, n (%)		
Hypertension	7 (53)	3 (43)
Type 2 diabetes mellitus	4 (31)	2 (29)
On statin	9 (69)	5 (71)
Body mass index >30 kg/m^2^	3 (23)	2 (29)
Aneurysm size, mm	60.8±11	…

Values are n (%) or mean ± standard deviation. AAA indicates abdominal aortic aneurysm.

**Table 2.** Patient Characteristics (Plasma Samples)

	Patients With AAA (n=23)	Patients With CAD (n=17)	Healthy Volunteers (n=12)
Age, y*	71.8±8.9*	70.8±9.0*	50.8±11
Male, n (%)	18 (78)	12 (71)	9 (75)
Background, n (%)			
Hypertension*	18 (78)	12 (71)	2 (17)
Type 2 diabetes mellitus	5 (22)	4 (24)	2 (17)
On statin*	18 (78)	13 (76)	3 (25)
Body mass index >30 kg/m^2^	5 (22)	6 (35)	3 (25)
Aneurysm size, mm	56.2±5.3	…	…

Values are n (%) or mean ± standard deviation.

For comparison of age, post hoc pairwise Student *t* test after 1-way analysis of variance was used, because age was considered to be normally distributed.

AAA indicates abdominal aortic aneurysm; CAD, coronary artery disease.

*Statistically significant difference (*P*<0.05).

### RNA Isolation and Purification From Tissue and Blood Samples

Aortic wall samples were submerged in RNAlater (Qiagen, Hilden, Germany) at 4°C and were homogenized by Tissue Lyser II homogenizer (Qiagen). We extracted total RNA from tissue and plasma samples by using the mirVana miRNA and mirVana PARIS RNA isolation kits, respectively (Ambion, Austin, TX). Concentration and purity of extracted RNA were evaluated by a spectrophotometer and stored at −80°C until analysis.

### miRNA Microarray

Total RNA preparations were reverse-transcribed with Megaplex Primer Pools (Human Pools A v2.1 and B v2.0), and miRNA expression was screened with TaqMan Human MicroRNA Array A (Applied Biosystems, Foster City, CA). We used an Applied Biosystems 7900HT thermal cycler and the manufacturer's recommended program to carry out quantitative PCR. Detailed analysis of the results was performed in the Real-Time Statminer Software (Integromics). The miRNA expression heat map was constructed by unsupervised hierarchical clustering.

### Real-Time PCR

Total RNA isolated from the stored specimens was reverse-transcribed with QuantiTect Reverse Transcriptase (Qiagen). Using the 7500 Fast Real-Time PCR System (Applied Biosystems), we performed real-time PCR assays for miRNAs; monocyte chemoattractant protein (MCP)-1; TNF-α; transforming growth factor (TGF)-β; interleukin-6 and -1β; matrix metalloproteinase-1, -2, -3, and -9; and tissue inhibitor of metalloproteinase-1, -2, -3, and -9. The miRNA assay values were normalized by expression of small nuclear RNA (miR-16), whereas values for other molecules were normalized by expression of glyceraldehydes-3-phosphate dehydrogenase.

### Histology

Aortic wall samples were fixed promptly in 10% formalin-phosphate buffer and embedded in paraffin. Five-micrometer sections were stained with hematoxylin and eosin or were immunohistolabeled with a mouse anti-CD68 monoclonal antibody (Dako, Glostrup, Denmark, 1:100 dilution) and visualized by the labeled streptavidin-biotin method (Dako). Histological slides were assessed by optical microscopy (BIOREVO BZ-9000, Keyence Corp, Osaka, Japan).

### Statistical Analysis

Continuous variables are summarized as mean ± standard deviation and categorical variables as frequencies and proportions. All the continuous variables were checked for normality via the Kolmogorov-Smirnov test and graphically. Normally distributed and nonnormally distributed variables were compared between 2 groups with the Student *t* test and the Mann-Whitney *U* test, respectively. Comparisons among 3 groups were made with the 1-way analysis of variance, followed by the post hoc pairwise Student *t* test, or the Kruskal-Wallis test, followed by the post hoc pairwise Wilcoxon-Mann-Whitney *U* test, as appropriate. Categorical variables were compared with the Fisher exact test. Correlations were tested with the use of the Spearman correlation coefficient. The multiple regression model also was used to adjust for the following baseline patient characteristics simultaneously: age, hypertension, and statin use. (There are statistically significant imbalances in these baseline characteristics among the 3 groups for plasma samples.) Nonnormally distributed dependent variables in the multiple regression analysis were natural log-transformed to satisfy normality of the used models, as appropriate. Multiple comparisons among the 3 groups in the multiple regression analysis were made with the use of the Tukey contrast described by Westfall^9^ and as implemented by Hothorn et al.^10^ The multiplicity in pairwise comparisons was corrected by the Bonferroni procedure. All probability values are 2 sided, and values of *P*<0.05 were considered to indicate statistical significance. Statistical analysis was performed in SPSS 15.0 (SPSS Inc, Chicago, IL) and the R program.^11^

## Results

### Differential Expression of miRNAs in Human AAA Tissue

**Figure 1.**
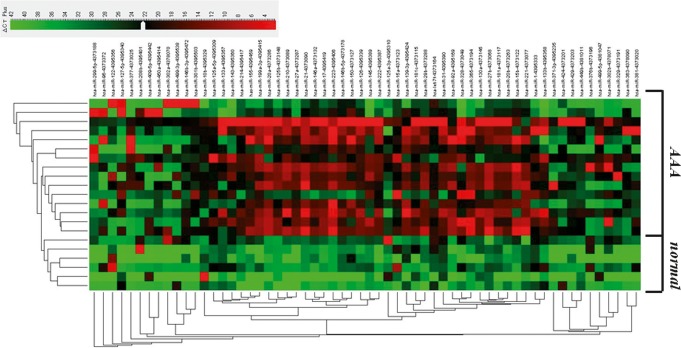
miRNA profiles distinguish the AAA group from the normal group. RNA was isolated from aortic wall tissue of patients with AAA (n=13) or normal aortic wall tissue (n=7). A heat map diagram is shown clustering the differentially expressed miRNAs. AAA indicates abdominal aortic aneurysm; miRNA, microRNA.

We comprehensively analyzed the miRNA expression profile in AAA wall tissue by using an miRNA microarray and compared it with the profile in normal aortic wall tissue. The heatmap diagram showed a marked difference in several miRNA expression levels (Figure 1). Among 384 miRNAs analyzed, 77 were upregulated >2-fold in AAA tissue, and none were downregulated compared to normal aortic tissue (Table 3).

**Table 3.** Upregulated miRNAs in Patients With AAA

miR	Aneurysm (2^−ΔCT^)	Normal (2^−ΔCT^)	Fold Change	*P*
let-7a	0.00045	9.91E-06	45.40502371	0.0018
let-7c	0.000172	6.38E-06	26.90076815	0.0018
let-7e	0.005	0.001	5	0.0039
let-7f	0.000172	3.27E-05	5.278910176	0.0018
miR-10a	0.000203	7.03E-06	28.82536974	0.0027
miR-10b	0.000399	1.71E-07	2329.638273	0.0018
miR-18a	2.33E-05	8.78E-07	26.5730199	0.0018
miR-20a	0.003036	0.000321	9.472324955	0.0018
miR-21	0.012678	0.000833	15.21774097	0.0018
miR-23b	0.000169	1.71E-07	985.4142357	0.0027
miR-25	0.000116	2.51E-06	46.11398964	0.0018
miR-27b	0.000368	1.54E-05	23.96091205	0.0027
miR-29b	7.03E-05	1.2E-06	58.58333333	0.0018
miR-30b	0.003	0.000448	6.693440428	0.0108
miR-32	6.77E-06	3.19E-07	21.22961104	0.0018
miR-92a	0.000334	9.54E-05	3.497903564	0.0018
miR-93	0.001	1.35E-05	74.23904974	0.0108
miR-99b	0.001	2.99E-05	33.50083752	0.0078
miR-101	5.99E-05	1.88E-06	31.8394471	0.0027
miR-103	0.000254	4.49E-05	5.650736264	0.0149
miR-107	5.4E-06	1.71E-07	31.50525088	0.0039
miR-124	1.66E-05	8E-07	20.75	0.0272
miR-126	0.113377	0.005061	22.40234294	0.0018
miR-127-3p	0.000111	6.36E-06	17.44186047	0.0039
miR-128	1.16E-05	1.99E-06	5.84876429	0.0361
miR-130b	6.62E-05	3.9E-06	16.96899821	0.0039
miR-132	0.001	6.58E-06	151.9525908	0.0039
miR-134	0.000128	5.67E-05	2.252512065	0.0202
miR-135a	5.17E-06	1.71E-07	30.15169195	0.0027
miR-135b	2.8E-05	1.2E-06	23.31109258	0.0202
miR-139-5p	0.001	6.22E-05	16.07200257	0.0078
miR-142-3p	0.003	4.38E-05	68.50879196	0.0018
miR-146a	0.12327	0.000677	182.0550879	0.0018
miR-146b-3p	1.14E-05	5.07E-07	22.5335438	0.0078
miR-148a	0.000279	2.16E-06	129.2264937	0.0018
miR-152	0.001	4.09E-05	24.44987775	0.0018
miR-181c	6.11E-06	6.11E-07	9.998850044	0.0149
miR-183	7.31E-06	1.71E-06	4.26546091	0.0039
miR-187	7.16E-06	1.71E-07	41.76779463	0.0039
miR-195	0.002465	0.00036	6.856867246	0.0039
miR-199a-5p	1.48E-05	1.71E-07	86.46441074	0.0039
miR-199a-3p	0.005	0.001	5	0.0039
miR-200b	2.19E-05	8.2E-07	26.7292912	0.0108
miR-210	0.001	7.35E-05	13.5998912	0.0055
miR-216a	1.02E-05	8.7E-07	11.71875	0.0108
miR-217	1.09E-05	4.99E-07	21.73042259	0.0039
miR-221	0.000225	8.4E-06	26.76190476	0.0149
miR-222	0.035285	0.002606	13.53771486	0.0018
miR-223	0.067432	0.001373	49.11975524	0.0018
miR-296-5p	1.09E-05	1.71E-07	63.71061844	0.0018
miR-301b	1.07E-05	1.71E-07	62.66044341	0.0018
miR-302c	9.77E-07	2.43E-07	4.014293176	0.0055
miR-324-5p	3.23E-05	5.14E-07	62.81602489	0.0018
miR-331-3p	0.003	0.001	3	0.0055
miR-155	0.004296	0.000851	5.048771889	0.0149
miR-342-5p	9.92E-06	8.22E-07	12.07081153	0.0039
miR-361-5p	1.5E-05	1.19E-06	12.6302521	0.0039
miR-374a	0.000428	5.41E-05	7.908738223	0.0027
miR-379	1.57E-05	2.19E-06	7.168344007	0.0149
miR-410	1.02E-05	2.82E-06	3.638126092	0.0474
miR-423-5p	4.66E-05	3.25E-06	14.32482313	0.0108
miR-425	0.001	3.37E-05	29.6912114	0.0474
miR-431	1.86E-05	1.71E-07	108.4014002	0.0027
miR-451	0.001	2.51E-06	398.089172	0.0018
miR-455-3p	4.17E-05	1.86E-06	22.37124464	0.0055
miR-495	2.59E-05	1.13E-06	22.98401421	0.0039
miR-502-3p	8.27E-06	2.35E-06	3.524296675	0.0202
miR-502-5p	6.95E-06	3.32E-07	20.93429777	0.0078
miR-532-3p	0.000188	1.4E-05	13.36182336	0.0078
miR-532-5p	0.001	3.32E-05	30.0932892	0.0027
miR-545	6.77E-06	3.37E-07	20.08607896	0.0027
miR-590-5p	0.001	1.21E-05	82.44023083	0.0018
miR-629	8.5E-06	1.71E-07	49.57409568	0.0055
miR-642	1.26E-05	1.01E-06	12.52234359	0.0018
miR-655	9.57E-06	4.9E-07	19.5187602	0.0108
miR-708	0.0003	4.78E-06	62.84518828	0.0018
miR-886-3p	0.001	8.38E-05	11.94029851	0.0027

AAA indicates abdominal aortic aneurysm.

**Figure 2.**
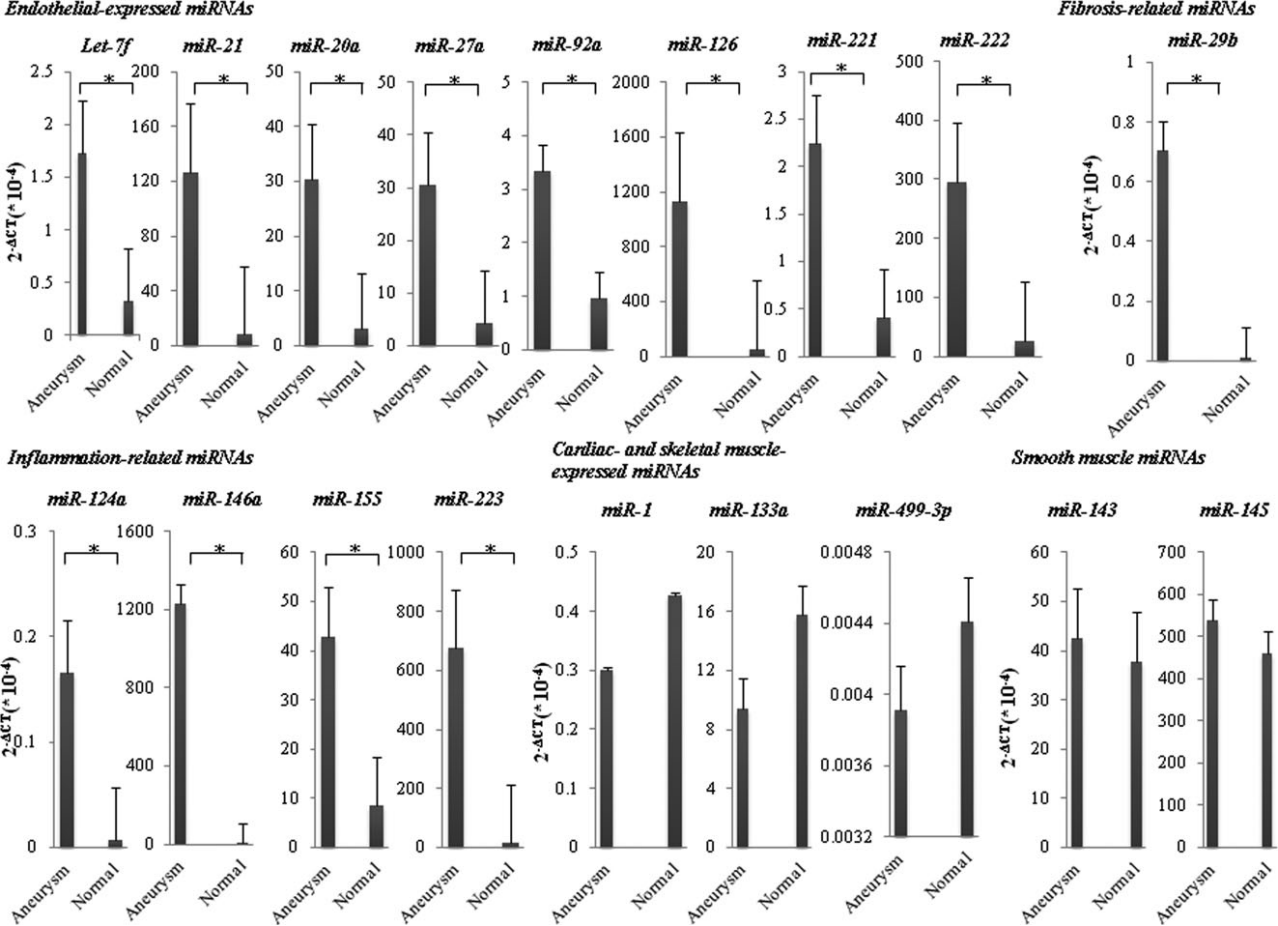
Validation of selected microarray data by real-time PCR. Assays were conducted in triplicate for each RNA sample, and the relative amount of each miRNA was normalized to U6 snRNA. *Statistically significant difference between AAA wall and normal aortic wall (*P*<0.05). Vertical bars represent standard error. AAA indicates abdominal aortic aneurysm; PCR, polymerase chain reaction.

The miRNAs that were reproducibly and reliably altered in the microarray analysis (*P*<0.05 and >2-fold change) were considered to be differentially expressed genes and were further assessed quantitatively by real-time PCR. Endothelial miRNAs, such as miR-126, miR-20a, miR-27a, miR-92a, miR-221, miR-222, members of the let-7 family, and miR-21, were significantly upregulated in AAA tissue compared with normal aortic tissue, whereas expression of vascular smooth muscle–enriched miRNAs, such as miR-143 and miR-145, were not significantly different (Figure 2). Cardiac- and skeletal muscle–expressed miRNAs, such as miR-1, miR-133a, and miR-499-3p, also showed no significant difference. Monocyte/macrophage–related miRNAs, such as miR-155, miR-146a, miR-223, and miR-124a, were significantly upregulated in AAA tissue compared with normal aortic wall tissue, as was the fibrosis-related miRNA, miR-29b (Figure 2).

### Inflammatory Changes in AAA Tissue

Inflammation-related miRNAs (miR-155, miR-146a, miR-223, and miR-124a) were upregulated in AAA tissue compared with normal aortic wall tissue; therefore, we further examined the severity of inflammation in AAA tissues.

**Figure 3.**
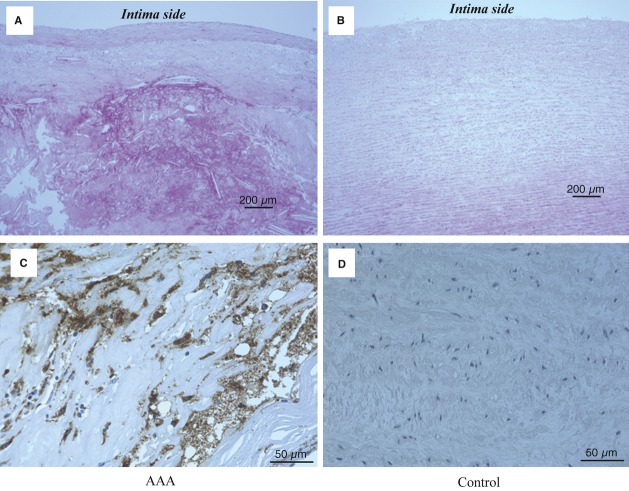
Representative hematoxylin-and-eosin staining and immunohistochemical sections of aorta show infiltration of inflammatory cells in AAA wall tissue (A and C) compared with normal aortas of controls (B and D). Presence and distribution of macrophages in the AAA tissues were assessed by immunohistolabeling for CD68 (C). Magnification: A and B, ×4 (hematoxylin-and-eosin staining); C and D, ×40 (immunostaining). AAA indicates abdominal aortic aneurysm.

Hematoxylin-and-eosin staining of AAA wall tissue showed typical features of established atherosclerosis, such as fibrotic changes of the media or inflammatory cell infiltration, in contrast to staining of normal aortic wall tissue, which showed 3 layers (adventia, media, and intima) and had a heterogeneous mixture of smooth muscle, endothelial cells, fibroblast cells, and a complex extracellular matrix (Figure 3A and 3B). We assessed macrophage presence and distribution in AAA tissues by immunohistolabeling for CD68. CD68-positive cells were abundantly present in the intima of AAA tissues compared to normal aortic wall tissue, in which CD68-positive cells were rarely present (Figure 3C and 3D).

### Correlation of Local miRNA Expression With Inflammatory Changes

The expression of specific miRNAs is altered by extracellular signals, such as MCP-1, cytokines (TNF-α, TGF-β), and fibroblast metalloprotease.^12–16^ miR-124a and miR-146a are key MCP-1 miRNAs, and miR-126, miR-146a, and miR-223 are associated with several inflammatory diseases.^12,15,17^

**Figure 4.**
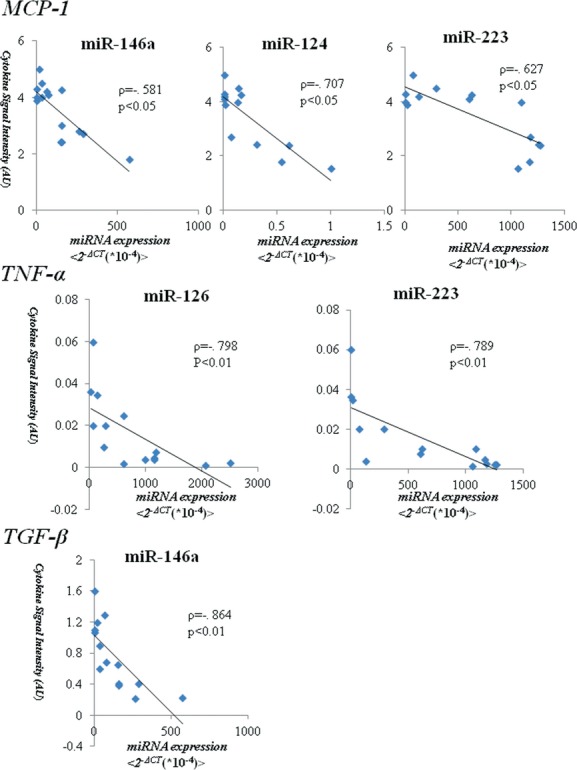
Correlation between miRNA and inflammatory cytokines in AAA tissue. AAA indicates abdominal aortic aneurysm; miRNA, microRNA.

We assessed expression of factors/molecules related to inflammation or fibrosis in AAA and normal aortic wall tissues by using real-time PCR for the following: MCP-1; TNF-α; TGF-β; matrix metalloproteinase-1, -2, -3, and -9; and tissue inhibitor of metalloproteinase-1, -2, -3, and -9. In addition, we statistically evaluated the correlation between expression of these factors and miRNAs. The results showed negative correlations between MCP-1 and miR-124, miR-146a, and miR-223; between TNF-α and miR-126 and miR-223; and between TGF-β and miR-146a (Figure 4).

### Plasma miRNA Signature in Patients With AAA

Subsequently, we analyzed preoperatively collected plasma of patients with AAA (n=23), patients with CAD (n=17), and healthy volunteers (n=12) by real-time PCR for the miRNAs that were upregulated in AAA tissues (miR-15a, -15b, -124a, -126, -223, -21, -29b, -155, and -146a). To determine an endogenous plasma control, we selected miR-16 and snRNA U6 RNAs as candidates. miR-16 was more stably expressed than U6 in our sample plasma and was therefore used as a housekeeping miRNA in this study.

**Figure 5.**
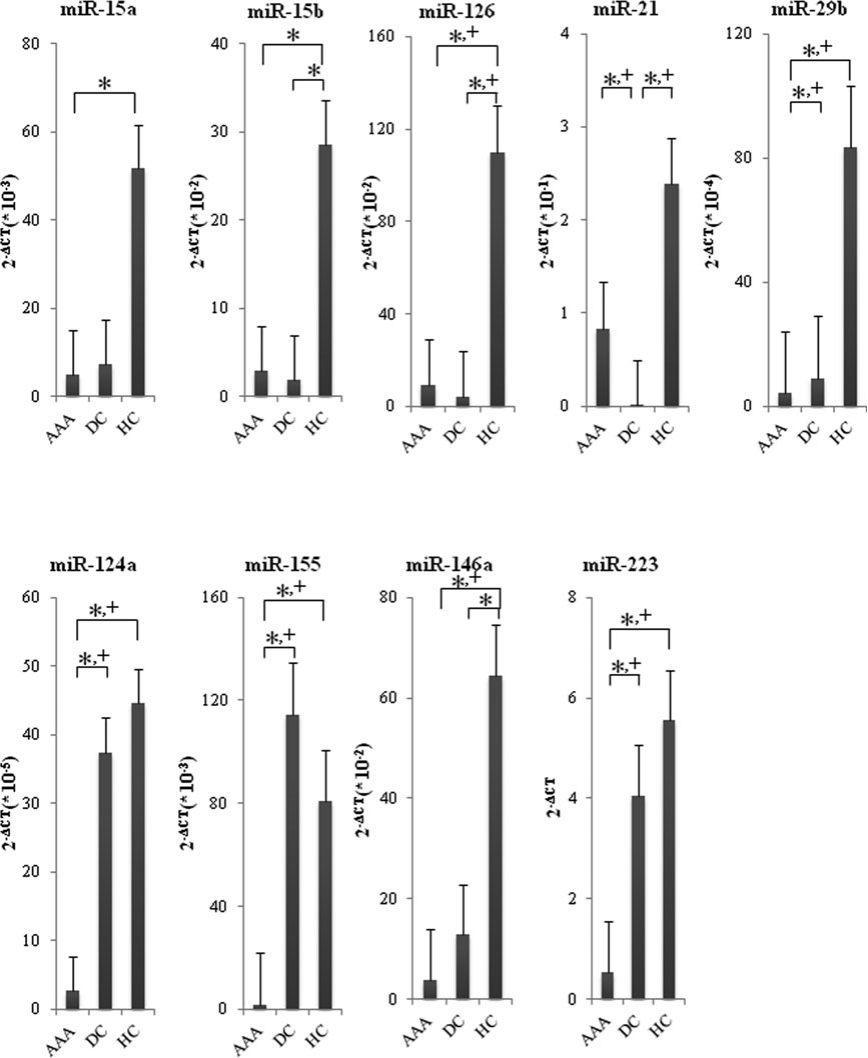
Circulating miRNAs in patients with AAA vs disease controls vs healthy volunteers. Expression of selected miRNAs in EDTA-plasma obtained from patients with AAA (n=23), disease controls (DC; n=17), and healthy controls (HC; n=12), as determined by TaqMan PCR. Post hoc pairwise Wilcoxon-Mann-Whitney *U* test was used after the Kruskal-Wallis test, and multiple regression analysis with natural log-transformation of the expression data was performed, because all the expression data were considered to be nonnormally distributed. Statistically significant differences: *P<0.05, multiple comparison with the Bonferroni correction based on the post hoc pairwise Wilcoxon-Mann-Whitney *U* test); +*P*<0.05, multiple comparison with the Bonferroni correction based on multiple regression analysis with adjustment for age, hypertension, and statin). Vertical bars represent standard error. AAA indicates abdominal aortic aneurysm; miRNA, microRNA; and PCR, polymerase chain reaction.

The healthy volunteers consistently expressed the miRNAs noted above in the plasma. In contrast, endothelial miRNAs (miR-126), inflammation-related miRNAs (miR-124a, -146a, -155, and -223), fibrosis-related miRNA (miR-29b), and apoptosis-related miRNA (miR-15a, -15b) were significantly downregulated in the plasma of patients with AAA compared to the plasma from healthy volunteers, according to the post hoc pairwise Wilcoxon-Mann-Whitney *U* test following the Kruskal-Wallis test. Moreover, inflammation-related miRNAs (miR-124a, -155, and -223) and fibrosis-ralated miRNA (miR-29b) were significantly downregulated in the plasma of patients with AAA compared to the plasma from patients with CAD (Figure 5).

However, there were statistically significant imblances in age, hypertension, and “on- statin” status among the 3 plasma sample groups (Table 2). Therefore, the multiple regression model also was used to adjust for these characteristics. As a result, in the adjusted analyses, expression of miRNAs such as fibrosis-related miRNA (miR-29b) and inflammation-related miRNAs (miR-124a, -155, and -223) was also significantly reduced in plasma of patients with AAA compared to healthy controls and patients with CAD (Figure 5). Moreover, in each of the adjusted analyses, the adjusted age, hypertension, and “on-statin” status become not significant.

## Discussion

We demonstrated that the miRNA expression profile in human AAA wall tissue was quite different from that in normal aortic wall tissue, including endothelial (let-7f and miR-20a, -21, -27, -92a, -126, -221, and -222), inflammatory (miR-124a, -146a, -155, and -223), and fibrosis-related miRNAs (miR-29b). In particular, we found that vessel wall–related and inflammatory cell–derived miRNAs were significantly upregulated in aortic aneurysm wall tissue. These miRNA expression changes were correlated closely with inflammatory cytokines, including MCP-1, TNF-α, and TGF-β, which are responsible for AAA pathophysiology. Moreover, we analyzed the circulating miRNA expression profile of patients with AAA. Interestingly, miRNAs that were upregulated in human AAA wall tissue were significantly downregulated in plasma from patients with AAA.

The characteristic pathological features of AAA are atherosclerotic change, chronic aortic wall inflammation, elastic media destruction, macrophage infiltration, neovascularization, and VSMC apoptosis.^17^ An inflammatory response to vascular injury can lead to atherosclerotic plaque formation, instigated by rapid proliferation, growth, and apoptosis of VSMCs, as well as aberrant VSMC plasticity that has been observed in a variety of pathological vascular disorders, including atherosclerosis. Increasing evidence indicates that numerous miRNAs, which could be responsible for the pathogenesis and pathophysiology of vascular injury and disease, show marked alterations in their expression.^5^ In our study, endothelial miRNAs including miR-21, miR-221, and miR-222 were significantly upregulated in AAA tissue compared to control tissue. These miRNAs are enriched in endothelial cells and regulate neointima lesion formation. Downregulation of aberrantly expressed miR-21 decreases neointima formation in rat carotid artery after angioplasty, which suggests that miR-21 regulates expression of some RNA expression responsible for neointimal lesion formation.^5^ At the molecular level, miR-21 controls VSMC proliferation and apoptosis via phosphatase, tensin homology deleted from chromosome 10 (PTEN),^5^ and programmed cell death 4 (PDCD4).^18^ PTEN is expressed in endothelial cells and VSMCs, where it modulates cell survival and apoptosis via its target molecules, phosphoinositide-3 kinases and Akt.^19^ PDCD4 attenuated by upregulated miR-21 induces VSMC apoptosis through upregulation of activator protein-1.^20–23^ In fact, a study showed that miR-21 was a key modulator of apoptosis of vascular wall smooth muscle cells during development of AAA in established murine models.^24^ We can speculate that upregulated miR-21 in AAA tissue induces VSMC apoptosis via downregulation of its 3 target genes and that measurement of miR-21 could become diagnostic in clinical settings.

A recent study showed that p27 (Kip1) and p57 (Kip2), negative regulators for VSMC proliferation, are 2 gene targets of miR-221 and miR-222 in rat carotid artery in vivo.^25^ Expression of miR-221 and miR-222 is upregulated and localized in the injured vascular walls of VSMCs in rat carotid arteries after angioplasty. Moreover, knockdown of miR-221 and miR-222 in rat carotid arteries suppressed VSMC proliferation in vivo and neointimal lesion formation after angioplasty.^26^ Thus, upregulation of tissue miR-21, -221, and -222 in the present study suggests that they are important factors for regulating VSMC apoptosis and proliferation and are involved in the pathogenesis of atherosclerotic diseases such as AAA, whereas the downregulation of these miRNAs in the circulation could predict pathological changes of the aortic wall.

There is also evidence that some miRNAs control vascular inflammation. miR-126 inhibits expression of vascular cell adhesion molecule-1, which mediates leukocyte adherence to endothelial cells.^26^ A decrease in endothelial cell miR-126 increases vascular cell adhesion molecule-1 expression and enhances leukocyte adherence. In addition, the myeloid-specific miR-223 regulates progenitor cell proliferation as well as granulocyte differentiation and activation during inflammation,^12^ and miR-146 is induced in macrophages by several microbial components and proinflammatory cytokines in a nuclear factor-κB–dependent manner.^2^ Thus, miR-126, miR-223, and miR-146 could be responsible for inflammatory responses via different pathways in various vascular diseases, including atherosclerosis and aortic aneurysm. Chronic inflammation of the aortic wall plays an important role in AAA pathogenesis, and activated macrophages are the primary cells secreting various proteases that lead to disruption of the orderly lamellar structure of the aortic media. Our study revealed that these inflammation-related and monocyte/macrophage–regulated miRNAs were significantly upregulated in AAA tissues and were significantly correlated with MCP-1, TNF-α, and TGF-β in diseased aortic walls, which suggests that these miRNAs indirectly predict the severity of aortic wall inflammation. Although these miRNAs are promising biomarkers of AAA or other atherosclerotic disease, inflammatory changes occur in other inflammatory diseases, such as rheumatism. These alterations in miRNA expression likely represent pathological rather than etiologic characteristics of AAA, and AAA should be carefully diagnosed in combination with the expression of other miRNAs.

In this study, miRNAs that were upregulated in AAA tissue were downregulated in the circulation. Tanaka et al^27^ have reported that plasma miR-92a values in non-Hodgkin's lymphoma were extremely low compared with healthy subjects, although miR-92a is overexpressed in malignant lymphoma cells. They speculated that miRNAs are packaged inside exsomes that are secreted from cells and that it might be possible that cancer cells specifically take in the exosome that contains miR-92a, with the result being that miR-92a decreases in the blood.^27–28^ As an alternative explanation, cancer cells might specifically digest miR-92a in the plasma directly or indirectly. Moreover, circulating levels of vascular and inflammation-associated miRNAs are significantly downregulated in patients with CAD,^29^ although tissue miRNA expression was not evaluated. There is speculation that miRNAs are delivered to atherosclerotic lesions by apoptotic bodies and that the reduction of circulating miRNAs in patients with CAD is caused by an uptake of circulating miRNAs into atherosclerotic lesions.^30^ In our study, we demonstrated that expression of many miRNAs was significantly upregulated in AAA wall tissue, which supports the above speculation, but further study is needed.

A limitation of the present study is that although our miRNA array analysis detected numerous miRNAs with significantly changed expression in AAA wall tissue, we did not confirm function. Further experimental studies are needed to explore unknown functions of other miRNAs that were expressed in AAA tissue and plasma. Additionally, we used thoracic aorta as control tissue. Although the developmental origin of thoracic aorta is the same as abdominal aorta, and miRNA expression was predicted to be similar, miRNA expression could vary slightly.

In summary, this study provides a first view of miRNA expression profiles in human AAA tissue and supports the idea that miRNAs play an important role in pathological processes of vascular diseases such as AAA. Moreover, circulating levels of vascular and inflammation-associated miRNAs that were upregulated in AAA tissue were significantly downregulated in plasma of patients with AAA. Analyses of miRNA expression could provide useful new biomarkers to diagnose AAA and some clues to develop miRNA-targeted AAA therapies.
